# Susceptibility of Urinary Tract Bacteria to Newer Antimicrobial Drugs

**DOI:** 10.3889/oamjms.2016.020

**Published:** 2016-01-25

**Authors:** Manjula Mehta, Jyoti Sharma, Sonia Bhardwaj

**Affiliations:** *Dr. Harvansh Singh Judge Institute of Dental Science & Hospital, Panjab University, Chandigarh, India*

**Keywords:** Antibiotic, Urinary tract infection, uropathogens

## Abstract

Urinary tract infections (UTIs) are among the commonest types of bacterial infections. The antibiotic treatment for UTIs is associated with important medical and economic implications. Many different microorganisms can cause UTIs though the most common pathogens are E. coli and members of family Enterobacteriaceae. The knowledge of etiology and antibiotic resistance pattern of the organisms causing urinary tract infection is essential. The present study was undertaken to evaluate trends of antibiotic susceptibility of commonly isolated uropathogens using newer antimicrobial agents, prulifloxacin, fosfomycin (FOM) and doripenem. We conclude that maintaining a record of culture results and the antibiogram may help clinicians to determine the empirical and/or specific treatment based on the antibiogram of the isolate for better therapeutic outcome.

## Introduction

Urinary tract infection (UTI) has long been recognized as one of the commonest bacterial infection [1] which is prevalent in both community and health care settings. Symptomatic infections are common and are associated with morbidity and rarely mortality. However asymptomatic infections are more common. Any demarcation regarding its prevalence in specific age group is not been reported. It may infect individuals from different age groups and from both the sexes [2]. It occurs as both complicated and uncomplicated infection, uncomplicated one is common in healthy adults and non-pregnant females whereas complicated UTI is frequently associated with structural and functional abnormalities [3]. Though the sufficient treatment options facilitate the management of the disease but increasing resistance towards various antimicrobial agents leads to numerous problems. Multi-drug resistant (MDR) uro-pathogens have leaded to increase in proportion of UTIs and because of limited treatment options for these MDR strains; it has become matter of serious concern and posing a financial burden too [4]. This study has been undertaken to evaluate the susceptibility of uro-pathogens to newer antimicrobial agents, prulifloxacin, fosfomycin (FOM) and doripenem.

Prulifloxacin is an oral fluoroquinolone specifically a lipophilic prodrug of ulifloxacin. It has broad spectrum antimicrobial activity against gram positive and gram negative bacteria as well [5]. The in vitro activity of prulifloxacin is generally greater than that of ciprofloxacin and other flouoroquinolones against isolates of Gram-negative bacteria, including *E. coli, Klebsiella* spp., *Proteus, Providencia* and *Morganella* spp., *Pseudomonas aeruginosa, Moraxella catarrhalis* and *Haemophilus* spp. Against Gram-positive bacteria, such as *Streptococcus* spp., *S. aureus, Enterococcus* spp. and coagulase-negative staphylococci [6], the in vitro activity of ulifloxacin is generally similar to or greater than that of ciprofloxacin, but lower than that of moxifloxacin [7]. FOM has shown promising *in vitro* activity against MDR uropathogens, however sufficient clinical data regarding its antimicrobial susceptibility behavior is not available. It is a phosphonic acid derivative and is a naturally occurring antibiotic. It acts primarily by interfering with bacterial peptidoglycan synthesis and hence disrupting cell wall synthesis [8]. It has been approved by Food and Drug Administration for the treatment of uncomplicated UTIs in women [9]. FOM represents its own class of antibiotics and no other member of this class is currently approved by regulatory agencies worldwide. It has got broad spectrum activity against both gram positive and gram negative bacteria. It can be administered orally with a convenient dosing schedule, including single dose therapy for uncomplicated cystitis. Doripenem a 1beta-methylcarbapenem, is a broad spectrum antibiotic which has been approved for intra-abdominal infections and complicated urinary tract infections [10]. The spectrum of activity of doripenem has been established *in vitro* and *in vivo* for gram negative, gram positive and anaerobic micro organisms. Compared with the other carbapenems, doripenem has a higher threshold for selection of non susceptible mutants in vitro, and it seems that high level resistance may require the coexistence of more than one resistance mechanism [11].

## Material and Methods

### Samples

This was a retrospective study with an observation period of eighteen months (May 2012 to October 2013) during which all the urine samples received in the department of Microbiology of Dr. Harvansh Sigh Judge Institute of Dental Sciences & Hospital were screened for the presence of bacteria. In this period of eighteen months the total number of samples screened was 259. Qualitative urine cultures were performed in CLED agar plates. Plates were incubated at 37°C for 18-24 h. Identification of the bacterial isolates was done using conventional biochemical methods [12].

### Antibiotic susceptibility testing

Besides the antibiotics viz amoxicillin, nitrofurantoin, ciprofloxacin, nalidixic acid, augmentin, amikacin, ceftazidime, cefotaxime, imepenem and sulbactam which are tested in routine; the sensitivity pattern of all the *E. coli* isolates was also tested for doripenem, fosfomycin and prulifloxacin. The Clinical and Laboratory Standard Institute (CLSI) criterion was used for the interpretation of the antimicrobial susceptibility [13].

## Result and Discussion

**Figure 1:**
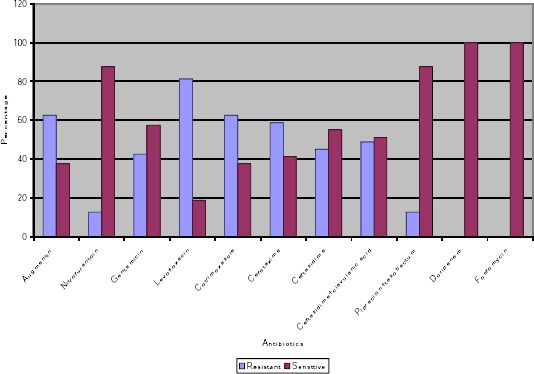
*Antibogram of E. coli isolates*.

Of all the samples received in microbiology department during the study period of eighteen months 30.8% (80) turned out to be *E. coli*. The susceptibility pattern of the eighty E. coli isolates tested for various antibiotics is described in Figure 1.

Out of eighty E. coli isolates fifteen (18.75%) were sensitive to prulifloxacin whereas number of isolates that were resistant to prulifloxacin were 65 (81.75%). If we look at the susceptibility of the E. coli isolates towards fluoroquinolones, this study showed that the different members of floroquinolones showed similar behavior. The finding is supported by another study by Carmignani et al. [14] who reported that there was no statistical significant difference in the clinical and microbiological parameters of prulifloxacin and ciprofloxacin. Other studies [6, 7] have shown that the ulifloxacin MICs and minimum bactericidal concentrations tend to be equal or even lower compared with ciprofloxacin, while they are generally lower compared with levofloxacin, for most gram-negative pathogens including *P. aeruginosa*. The strains of E. coli were altogether sensitive to doripenem and fosfomycin as our study reported 100% susceptibility to both these drugs. Our study was supported by another study by Maraki [15] which indicated acivity of fosfomycin against a considerable percentage of urinary isolates that simultaneously exhibited high rates of antimicrobial drug resistance to the conventionally used antimicrobial agents for the treatment of UTIs. The excellent activity of fosfomycin against E. coli has been reported by similar susceptibility findings [16] indicating that the drug is a valuable therapeutic option for urinary tract infections. In vitro activity of doripenem and other antimicrobial agents was evaluated against Gram-negative bacilli recently isolated in Brazilian study [17] and these studies supported our finding of 100 percent susceptibility of uropathogens to doripenem. Hence like other newer drugs, doripenem exhibited excellent urinary bactericidal activity and appears to be a good alternative in the empirical treatment of UTI and pyelonephritis. This study highlighted that susceptibility pattern is necessary to obtain sensitivity reports before start of antibiotic treatment in cases of suspected UTI. The knowledge of antimicrobial pattern of routinely isolated uropathogens may provide guidance to clinicians regarding the empirical treatment of UTI when therapy must be started before laboratory reports are available
